# Melanoma-specific mortality and competing mortality in patients with non-metastatic malignant melanoma: a population-based analysis

**DOI:** 10.1186/s12885-016-2438-3

**Published:** 2016-07-07

**Authors:** Weidong Shen, Naoko Sakamoto, Limin Yang

**Affiliations:** Department of Otolaryngology - Head and Neck Surgery, Chinese PLA General Hospital, The Institute of Otolaryngology, 28 Fuxing Road, Beijing, 100853 People’s Republic of China; Department of Epidemiology Research, Toho University, 4-16-20, Omori-Nishi Ota-ku, Tokyo, 143-0015 Japan; Division of Allergy, Department of Medical Subspecialties, National Center for Child Health and Development, 2-10-1 Okura, Setagaya-ku, Tokyo, 157-8535 Japan; Medical Support Center for Japan Environment and Children’s Study, National Center for Child Health and Development, 2-10-1 Okura, Setagaya-ku, Tokyo, 157-8535 Japan

**Keywords:** Censoring, Competing risks, Cumulative incidence, Prediction model, Melanoma

## Abstract

**Background:**

The objectives of this study were to evaluate and model the probability of melanoma-specific death and competing causes of death for patients with melanoma by competing risk analysis, and to build competing risk nomograms to provide individualized and accurate predictive tools.

**Methods:**

Melanoma data were obtained from the Surveillance Epidemiology and End Results program. All patients diagnosed with primary non-metastatic melanoma during the years 2004–2007 were potentially eligible for inclusion. The cumulative incidence function (CIF) was used to describe the probability of melanoma mortality and competing risk mortality. We used Gray’s test to compare differences in CIF between groups. The proportional subdistribution hazard approach by Fine and Gray was used to model CIF. We built competing risk nomograms based on the models that we developed.

**Results:**

The 5-year cumulative incidence of melanoma death was 7.1 %, and the cumulative incidence of other causes of death was 7.4 %. We identified that variables associated with an elevated probability of melanoma-specific mortality included older age, male sex, thick melanoma, ulcerated cancer, and positive lymph nodes. The nomograms were well calibrated. C-indexes were 0.85 and 0.83 for nomograms predicting the probability of melanoma mortality and competing risk mortality, which suggests good discriminative ability.

**Conclusions:**

This large study cohort enabled us to build a reliable competing risk model and nomogram for predicting melanoma prognosis. Model performance proved to be good. This individualized predictive tool can be used in clinical practice to help treatment-related decision making.

## Background

In the United States, there were an estimated 76,690 new melanoma patients in 2013, causing approximately 9480 deaths [1]. At the time of diagnosis, a large proportion of patients are diagnosed with localized disease [2]. According to data from the Surveillance, Epidemiology, and End Results (SEER) program of the National Cancer Institute, the 5-year overall survival rate for patients with melanoma diagnosed between 2004 and 2012 was 81 %, and for those with tumor size smaller than 1 mm, which constitutes approximately 65 % of all newly diagnosed melanomas, the outcomes are excellent, with a 5-year survival rate of 89 % [3]. The majority of patients with melanoma are cured by adequate surgical excision [4]. Given this situation, many patients may survive longer and eventually die from non-cancer-related causes. Hence, competing causes of death should be taken into account when evaluating the prognosis for patients with melanoma. Moreover, the probabilities of melanoma-specific mortality and competing risk mortality are valuable when planning treatment and follow-up regimens. However, these issues involving competing risk analysis have not yet been well described for melanoma.

Therefore, the objectives of this study were to evaluate and model the probability of melanoma-specific death and competing causes of death for patients with melanoma by competing risk analysis, and to build competing risk nomograms to provide individualized and accurate predictive tools using the SEER database, a large population-based cohort.

## Methods

Melanoma data were obtained from the National Cancer Institute’s SEER program, Public Use Data, for the period of 1973–2012 [3]. All patients with a diagnosis of primary non-metastatic melanoma in the SEER-18 registries during the years 2004–2007 were potentially eligible for inclusion in our study cohort. The SEER-18 registries cover approximately 28 % of the US population and includes the registries of San Francisco (SF) - Oakland, Connecticut, Detroit (Metropolitan), Hawaii, Iowa, New Mexico, Seattle (Puget Sound), Utah, Atlanta (Metropolitan), San Jose-Monterey (SJM), Los Angeles (LA), Alaska Natives, Rural Georgia, California excluding SF/SJM/LA, Kentucky, Louisiana, New Jersey, and Greater Georgia. Institutional review board approval and informed consent were not required in current study because SEER Research Data is publicly available and all patient data are de-identified. All authors have signed authorization and received permission from SEER to access and use the dataset.

**Fig. 1:**
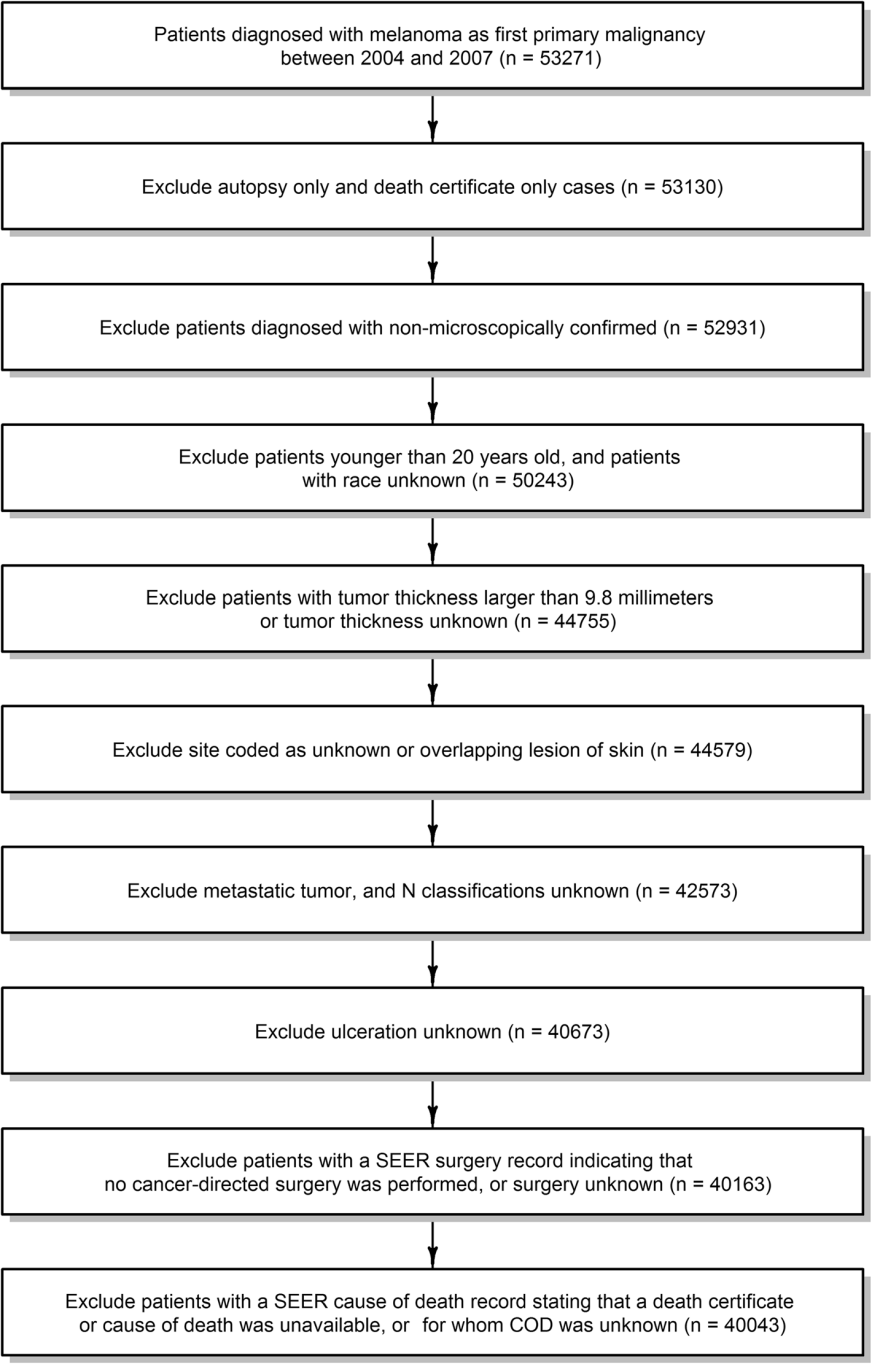
Flow chart of data selection

The study cohort consisted of patients with the following *International Classification of Disease for Oncology*, Third Edition (ICD-O-3), histology codes: 8720–8723, 8728, 8730, 8740–8746, 8761, 8770–8774, and 8780; and the ICD-O-3 site code C440–449. Only histologically confirmed malignant melanoma cases were included. Cancers diagnosed at autopsy or by death certificate only were excluded. Additional patients were also excluded for the following reasons: age, race, thickness, ulceration, site, and N stage classified as unknown; tumor thickness >9.8 mm; site coded as overlapping lesion of skin; cancer diagnosed as metastatic tumors; and being younger than 20 years old. We further excluded those with SEER surgical codes indicating that no cancer-directed surgery had been performed, or it was unknown whether cancer-directed surgery had been performed. Finally, death cases with an SEER cause of death record indicating that a death certificate was unavailable or was available, but without information on the cause of death, were excluded from the final cohort. The detailed data selection process and criteria are shown in Fig. 1. After data selection, our final study cohort included 40,043 cases diagnosed between 2004 and 2007, and followed up through 2012.

The cumulative incidence of death is presented by individual characteristics, as well as other clinical and pathologic factors. Age was classified into three groups (20–39 years, 40–64 years, and over 65 years). Tumor thickness, node, and ulceration were divided according to American Joint Committee on Cancer (AJCC) classification as follow: tumor thickness (≤1.00 mm, 1.01–2.00 mm, 2.01–4.00 mm, and >4.00 mm); ulceration (absent vs. present), and lymph node status (N0, N1, N2, and N3). We also presented prognosis separately for patients with stage I/II disease and stage III disease. Histological subtype included superficial spreading melanoma, nodular melanoma, lentigo maligna melanoma, malignant melanoma, not otherwise specified (NOS), and other melanoma. Anatomic site was grouped as extremities, trunk, face and ears, and scalp and neck.

Patients in the cohort were followed for vital status until the earliest of the following dates: death; last contact if before December 31, 2012; or December 31, 2012, if the date of last contact was after 2012. Death from melanoma and death from other causes were two event types in the competing risk analysis. The information regarding the cause of death came from death certificates. The cumulative incidence function was used to describe the probability of melanoma mortality and competing risk mortality. We used Gray’s test to compare differences of CIF between groups [5]. The proportional subdistribution hazard approach by Fine and Gray was used to model CIF [6]. Unlike a cause-specific hazards modeling approach, which requires modeling of both the event of interest and competing risk events to calculate CIF, in a subdistribution proportional hazards model, the predicted risk at a specific time point is calculated based on the cumulative subdistribution baseline hazard and the estimates of the regression coefficients from the model. Therefore, covariates in the fitted model can be incorporated into a nomogram easily [7, 8]. The exp (β) presents the increase of the hazard of subdistribution owing to a one unit crease of covariate x. A practical introduction on competing risks analysis can also be found in Pintilie’s book [9]. Variables used for modeling included age, tumor thickness, sex, race, histological subtype, anatomic site, ulceration, and lymph node status. Although over 40 % of the patients were diagnosed as having malignant melanoma, NOS, we included it when developing models because previous studies identified histological subtype as an independent prognostic factor for patients with melanoma. Moreover, other published prognostic models for melanoma using SEER data did not exclude histological subtype. To be consistent, in this study, histological subtype was included for modeling. When building predictive nomograms, the study cohort was randomly divided into training data (67 %) and validation data (33 %). A total of 26,829 cases as training data were used for building the model, and 13,214 cases were used as a validation dataset for evaluating model performance. The restricted cubic splines with three knots at the 10, 50, and 90 % empirical quantiles were fitted to model the variables of age at diagnosis and tumor thickness, which were treated as continuous variables in the model. The restricted cubic splines approach is a method used in the modeling process to relax linear assumptions for continuous predictors. Function and more detailed explanation can be found in Harrell’s book with regard to modeling strategy [10]. The Bayesian information criterion was used for model selection. The proportional hazards assumption was examined graphically with the plots of Schoenfeld-type residuals against time failure for each variable in the model. Finally, we built competing risk nomograms based on the models that we developed.

Both discrimination and calibration were evaluated to assess model performance. We used an index of probability of concordance between predicted probability and response (c-index) to quantify discrimination. The c-index can be defined as the proportion of all “evaluable ordered patient pairs” for which the patients who died first had the worse predicted outcome from the model [11]. A total of 200 bootstraps were used to generate the confidence interval for the c-index. To plot the calibration curve, the validation cohort was divided into quintiles according to predictions of probability of mortality. Subsequently, the observed CIF was calculated for each quintile. We then plotted the predictions of probability of mortality on the x-axis and the observed CIF on the y-axis to form a calibration curve. For a model that cali`brates well, the dots in the calibration curve are located close to a 45° diagonal line.

Statistical analyses were carried out with R version 3.1.0 software (Institute for Statistics and Mathematics, Vienna, Austria; www.r-project.org) [12]. The R package rms [13] and cmprsk [14] were used for building the model and nomogram. An R function provided by Wolbers was used to calculate the c-index of the competing risk model [11]. All *P* values were calculated using two-sided statistical testing.

## Results

Characteristics of the patient cohort are listed in Table 1. The cohort included 40,043 patients. In the whole cohort, 14.8 % of patients were aged 20–39 years, 51.9 % were aged 40–64 years, and 33.3 % were aged 65 years or older. The majority of patients were male (55.7 %) and white (98.8 %). Malignant melanoma, NOS (47.3 %) was the most common histological subtype, followed by superficial spreading melanoma (33.6 %), nodular melanoma (7.2 %), lentigo maligna melanoma (6.0 %), and other melanomas (6.0 %). Approximately, 46.0 % of melanomas occurred in the extremities, followed by 34.9, 12.0, and 7.0 % that were found in the trunk, face and ears, or scalp and neck. A total of 68.1 % of patients had tumors smaller than 1 mm. Ulceration was present in 12.4 % of patients and 6.7 % had positive lymph node involvement.

**Table 1:** Five-year cumulative incidences of death among patients with melanoma

			Cause-specific death		Death from other causes	
Characteristics	N (%)	Event	5-year (%)	*P*	5-year (%)	*P*
Total	40043	7216	7.1 (6.8 to 7.3)		7.4 (7.1 to 7.6)	
Age (years)				< 0.001		< 0.001
20–39 years	5940 (14.8)	262	3.3 (2.8 to 3.7)		0.4 (0.3 to 0.6)	
40–64 years	20766 (51.9)	2069	5.7 (5.4 to 6.0)		2.3 (2.1 to 2.5)	
65+ years	13337 (33.3)	4885	10.8 (10.3 to 11.3)		18.2 (17.5 to 18.9)	
Sex				< 0.001		< 0.001
Male	22295 (55.7)	4901	8.7 (8.3 to 9.1)		8.9 (8.5 to 9.3)	
Female	17748 (44.3)	2315	5.0 (4.7 to 5.3)		5.4 (5.1 to 5.8)	
Race				< 0.001		0.006
White	39559 (98.8)	7075	6.9 (6.6 to 7.1)		7.4 (7.1 to 7.7)	
Non-white	484 (1.2)	141	20.7 (17.2 to 24.4)		4.2 (2.7 to 6.3)	
Melanoma subtype				< 0.001		< 0.001
Superficial spreading melanoma	13444 (33.6)	1645	3.9 (3.6 to 4.2)		5.4 (5.0 to 5.8)	
Nodular melanoma	2875 (7.2)	1233	25.6 (24.0 to 27.2)		12.1 (10.9 to 13.3)	
Lentigo maligna melanoma	2383 (6.0)	547	3.4 (2.7 to 4.2)		14.5 (13.1 to 16.0)	
Melanoma, NOS	18926 (47.3)	3131	6.2 (5.9 to 6.5)		6.9 (6.5 to 7.3)	
Others	2415 (6.0)	660	12.8 (11.5 to 14.2)		9.2 (8.1 to 10.4)	
Anatomic sites				< 0.001		<0.001
Extremities	18427 (46.0)	2811	5.9 (5.6 to 6.3)		6.2 (5.9 to 6.6)	
Trunk	13987 (34.9)	2250	6.9 (6.5 to 7.3)		5.9 (5.5 to 6.3)	
Face and ears	4819 (12.0)	1331	8.0 (7.2 to 8.8)		13.8 (12.8 to 14.8)	
Scalp and neck	2810 (7.0)	824	13.9 (12.6 to 15.2)		10.8 (9.6 to 11.9)	
Thickness (mm)				< 0.001		< 0.001
≤ 1.00	28017 (70.0)	3086	2.2 (2.0 to 2.4)		5.9 (5.7 to 6.3)	
1.01–2.00	6279 (15.7)	1415	9.4 (8.7 to 10.2)		8.1 (7.5 to 8.8)	
2.01–4.00	3464 (8.9)	1391	21.2 (19.9 to 22.6)		11.8 (10.8 to 12.9)	
> 4.00	2283 (5.7)	1324	38.1 (36.1 to 40.1)		15.2 (13.7 to 16.7)	
Ulceration				< 0.001		< 0.001
Absent	35065 (87.6)	4916	4.1 (3.9 to 4.3)		6.6 (6.3 to 6.8)	
Present	4978 (12.4)	2300	27.6 (26.4 to 28.9)		12.8 (11.9 to 13.8)	
Lymph node status				< 0.001		< 0.001
N0	37363 (93.3)	5889	4.8 (4.6 to 5.0)		7.4 (7.1 to 7.7)	
N1	1543 (3.9)	650	31.0 (28.7 to 33.4)		6.0 (4.9 to 7.3)	
N2	762 (1.9)	401	38.8 (35.5 to 42.3)		7.8 (6.0 to 9.9)	
N3	375 (0.9)	276	63.8 (58.7 to 68.5)		6.2 (4.0 to 8.9)	
Stage				<0.001		< 0.001
I/II	37363 (93.3)	5889	4.8 (4.6 to 5.0)		7.4 (7.1 to 7.7)	
III	2680 (6.7)	1327	37.8 (36.0–39.7)		6.5 (5.7–7.5)	

*Abbreviation*: *NOS* malignant melanoma, not otherwise specified

**Fig. 2:**
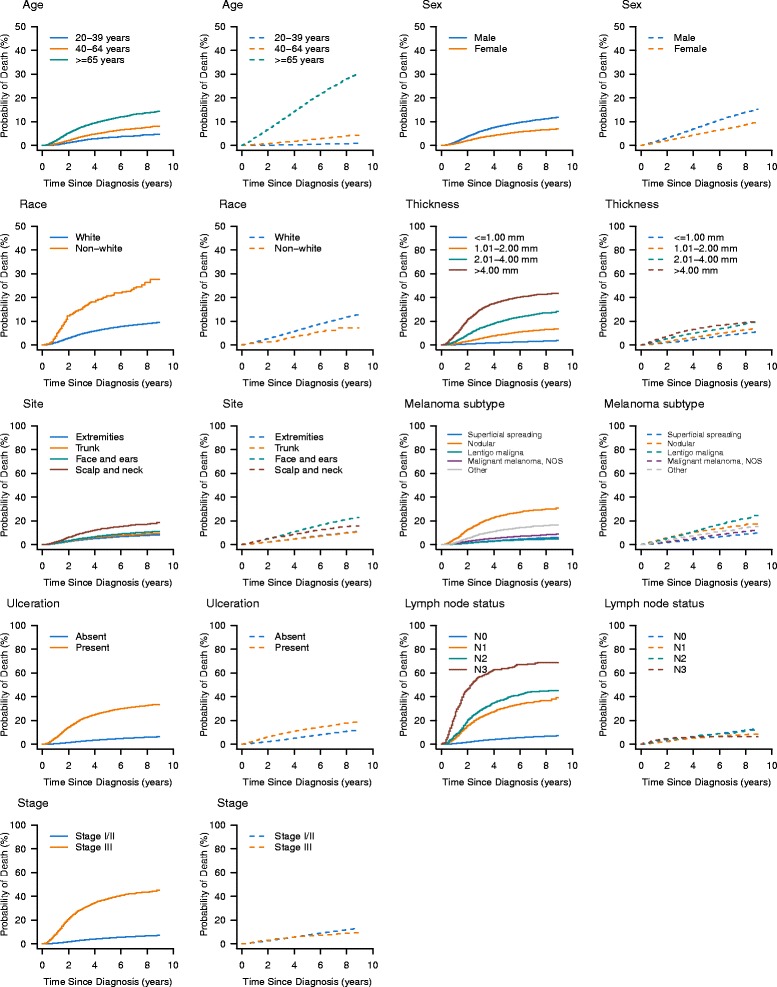
Cumulative incidence estimates of death by patient characteristics (solid line, melanoma death; dotted line, non-melanoma death)

The median follow-up was 76 months (interquartile range, 63–90). A total of 7216 patients died during the follow-up period, of whom 3304 died from melanoma and 3912 died owing to causes other than melanoma. Of the 3912 patients who died of causes other than melanoma, the most common causes of competing mortality were diseases of the heart (29.3 %), cerebrovascular diseases (6.9 %), and lung and bronchus tumors (6.3 %). Five-year estimates of the crude cumulative incidence of death from melanoma and other causes by individual characteristics, as well as clinical and pathologic factors, are presented in Table 1. The 5-year cumulative incidence of melanoma death was 7.1 % (95 % confidence interval [CI], 6.8–7.3) and the cumulative incidence of other causes of death was 7.4 % (95 % CI, 7.1–7.6). CIF curves are plotted in Fig. 2. The 5-year cumulative probability of death from melanoma increased with increasing age at diagnosis. The 5-year CIF for other causes of death also increased with increasing age. The probability of death from melanoma was significantly greater in male than in female patients. Non-white patients were more likely to die as a result of melanoma, and less likely to die as a result of other causes than those of white patients. Patients with nodular melanoma had a poor prognosis, with a 25.6 % 5-year cumulative probability of melanoma death and a 12.1 % 5-year probability for other causes of death. Compared with melanoma that occurred in the extremities and trunk, melanoma located in the head and neck had a greater probability of death from melanoma, and also a greater probability of death from other causes. Both probability of melanoma-specific death and other causes of death increased with increasing tumor thickness. Patients with ulcerated disease had a poor prognosis, with a 27.6 % 5-year cumulative probability of melanoma death. Patients with positive node (stage III disease) were more likely to die from melanoma than those with negative node (stage I/II disease).

Coefficients and subdistribution hazard ratios (_sd_HR) from the multivariable analysis are presented in Table 2. Proportional subdistribution hazard assumption was held for variables used for modeling. Age was strongly predictive of melanoma-specific mortality. Increasing tumor thickness was related to an increased probability of death from melanoma. Race was a significant independent predictor for time of melanoma death, with a significant _sd_HR of 1.82 (95 % CI, 1.42–2.32) for non-white patients, compared with white patients. Patients with nodular melanoma were more likely to die of melanoma than those with superficial spreading melanoma, with a _sd_HR of 1.41 (95 % CI, 1.21–1.63). The anatomic site was a significant independent predictor of melanoma death. In addition, patients that presented with ulceration disease were more likely to die of melanoma with _sd_HR of 1.98 (95 % CI, 1.72–2.22). Positive node was associated with an increasing probability of melanoma death, with _sd_HR of 2.86 (95 % CI, 2.49–3.29), 3.41 (95 % CI 2.88–4.04), and 5.69 (95 % CI 4.54–7.12) for N1, N2, and N3 disease, respectively, compared with N0. The probability of death from other causes was also modeled. Older patients, male, white race, and negative lymph node involvement were associated with a higher likelihood of death from non-melanoma causes.

**Table 2:** Proportional subdistribution hazards models of probabilities of death

	Cancer-specific mortality			Other causes of mortality		
Characteristics	Coefficient	_sd_HR (95 % CI)	*P*	Coefficient	_sd_HR (95 % CI)	*P*
Age	0.018	-	< 0.001	0.06	-	< 0.001
Age’	0.001	-	0.85	0.032	-	< 0.001
Thick	1.71	-	< 0.001	-	-	-
Thick’	−3.659	-	< 0.001	-	-	-
Female	−0.234	0.79 (0.72 to 0.87)	< 0.001	−0.359	0.70 (0.64 to 0.76)	< 0.001
Non-white	0.601	1.82 (1.43 to 2.32)	< 0.001	−0.583	0.56 (0.34 to 0.92)	0.02
Melanoma subtype	-	-	-	-	-	-
Nodular melanoma	0.341	1.41 (1.21 to 1.63)	< 0.001	-	-	-
Lentigo maligna melanoma	−0.114	0.89 (0.68 to 1.17)	0.41	-	-	-
Malignant melanoma, NOS	0.213	1.24 (1.10 to 1.40)	< 0.001	-	-	-
Other melanomas	0.272	1.31 (1.10 to 1.57)	< 0.001	-	-	-
Anatomic sites	-	-	-	-	-	-
Trunk	0.23	1.26 (1.13 to 1.40)	< 0.001	-	-	-
Face and ears	0.277	1.32 (1.14 to 1.52)	< 0.001	-	-	-
Scalp and neck	0.503	1.65 (1.41 to 1.94)	< 0.001	-	-	-
Ulceration (present)	0.683	1.98 (1.77 to 2.22)	< 0.001	-	-	-
Lymph node status	-	-	-	-	-	-
N1	1.051	2.86 (2.49 to 3.29)	< 0.001	−0.201	0.82 (0.64 to 1.05)	0.11
N2	1.228	3.41 (2.88 to 4.04)	< 0.001	−0.16	0.85 (0.62 to 1.14)	0.29
N3	1.739	5.69 (4.54 to 7.12)	< 0.001	−0.58	0.56 (0.33 to 0.95)	0.03

*Abbreviations*: _*sd*_ *HR* subdistribution hazard ratio

**Fig. 3:**
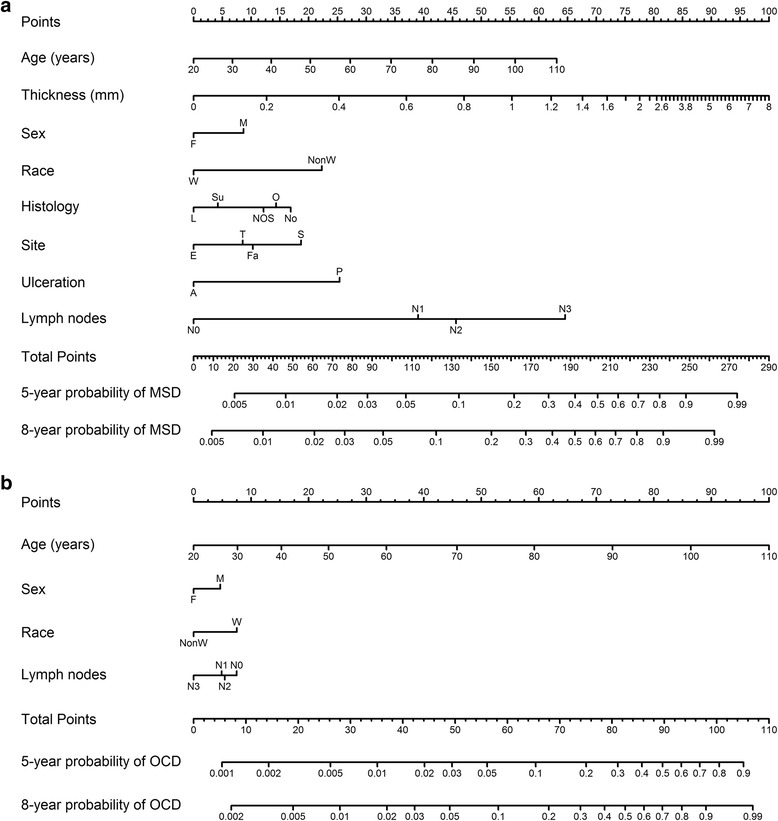
Nomogram for predicting 5- and 8-year probabilities of mortality in patients with melanoma. Abbreviations: Sex: F, female; M, male; Race: W, white; NonW, non-white; Histology: Su, superficial spreading melanoma; No, nodular melanoma; L, lentigo maligna melanoma; NOS, malignant melanoma, not otherwise specified; O, other melanomas; Site: E, extremities; T, trunk; F, face and ears; S, scalp and neck; MSD, melanoma-specific death; OCD, other causes of death. Instructions: Locate the patient’s characteristic on the variable row, and draw a vertical line straight up to the points’ row to obtain a value of points for the variable. Repeat this process, and assign points for each variable. Add up the total points and draw a vertical line from the total points’ row to obtain the probability of mortality ((**a**), melanoma-specific death; (**b**), other causes of death)

**Fig. 4:**
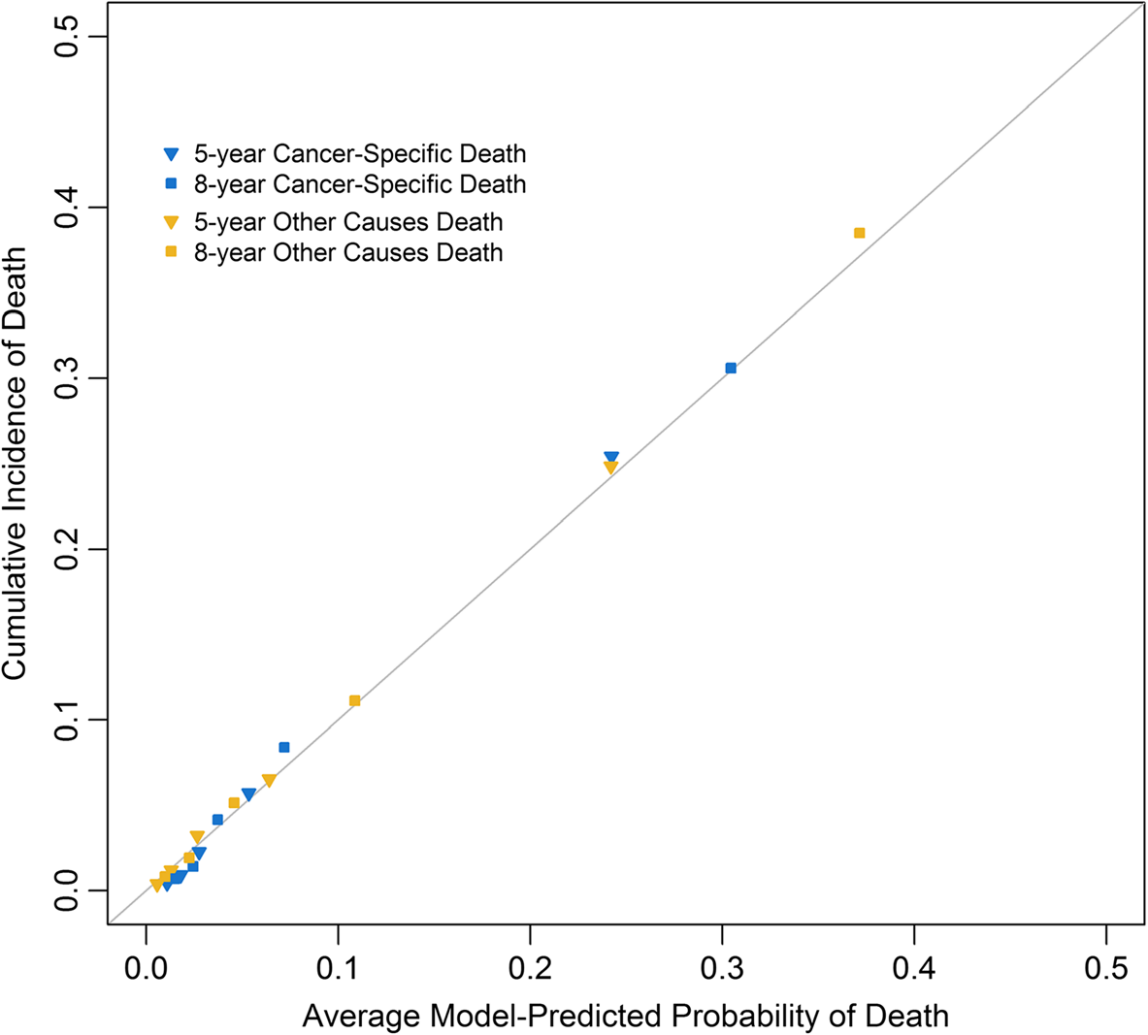
Calibration plot. The X-axis designates the mean predicted probability of mortality based on the model. The Y-axis indicates the observed cumulative incidence of death. The solid line represents equality between the predicted and observed values

The nomograms based on models that we developed are shown in Fig. 3. To use the nomogram, first, locate the patient’s characteristic on the variable row, and draw a vertical line straight up to the points’ row to obtain a value of points for the variable. For example, for a patient aged 50 years, if a vertical line is drawn straight up to the “Point” row, we got around 20 points. Then, repeat the process for each row and assign points for each variable. Add up the total points and draw a vertical line from the total points’ row to obtain the probability of mortality. For example, if the sum of points for each variables was “90”, the corresponding “5-year probability of melanoma-specific death” would be a 5-year probability of melanoma-specific death of 3.1 %. The calibration plot is shown in Fig. 4. The calibration plot indicates that the nomograms were well calibrated because the predicted probability of mortality and the actual CIF were in good agreement. C-indexes were 0.85 (95 % CI, 0.84–0.86) and 0.83 (95 % CI, 0.82–0.84) for nomograms predicting the probability of melanoma mortality and competing risk mortality, which suggests good model discriminative ability.

## Discussion

In this study, we estimated that the 5-year probability of death for patients with non-metastatic melanoma diagnosed between 2004 and 2007 were 7.1 and 7.4 % for cause-specific mortality and competing mortality, respectively. Of the 7261 deaths in the study cohort, 3912 (54 %) were owing to causes other than melanoma. We built nomograms to serve as comprehensive and easily used clinical tools that can predict the probability of melanoma-specific mortality and mortality from other causes.

We found that older patients were more likely to die of melanoma. These results are consistent with other published studies. For example, Balch et al. used a cohort of 11,088 melanoma patients from the expanded American Joint Committee on Cancer melanoma staging database to evaluate survival among patients with melanoma. They found primary melanoma became more advanced with increasing age. Moreover, older patients with melanoma were more likely to have a disease with a thicker tumor, higher mitotic rate, and were more like to be ulcerated [15]. In addition, older patients were found to be more likely to have age-related comorbid conditions that prevent them from receiving the same standards of care that are provided for younger patients [16].

Other characteristics associated with an elevated probability of melanoma mortality included: male, non-white, thick tumor, present ulceration, nodular melanoma, head and neck melanoma, and positive node status. Similar results were also reported by Marashi-Pour et al. [17]. They studied 52,330 invasive melanomas in New South Wales using a competing risk method, and found that older patients, male patients, patients with thick tumors, non-localized disease, nodular melanoma, and patients with head neck melanoma showed a high risk of melanoma mortality [17].

The prognostic nomogram is a model-based prediction tool that incorporates clinical and pathologic risk factors known to have an impact on outcome [18, 19]. Although the TNM staging system has significant predictive capability for the prognosis of patients with melanoma, some important risk factors, such as age, are not included. As mentioned above, age is an independent predictive factor of melanoma mortality. Without considering age, regarding all patients within the same TNM category as a homogeneous group may lead to a bias in estimating the prognosis. The nomograms described here not only include tumor thickness, node status, and ulceration that are used for AJCC classification, but also incorporate demographic characteristics. Moreover, different from a scoring system, a nomogram provides a quantified prognosis for individual patients, so it is more acute and informative.

Estimating prognosis based on individual risk profiles is important for patient counseling and decision making. For example, in clinical practice, the prediction of prognosis and risk classification can help clinicians to devise treatments and make a follow-up plan. In addition, it can be used in clinical studies to identify and select the appropriate patient population based on predicted prognosis, and it can help to create subgroups for comparing the effectiveness of a treatment within each subgroup. In addition, predicted prognosis can also be incorporated into a multivariate model as an adjusting factor when evaluating different treatment strategies [20].

Melanoma is a cancer with a good prognosis, having a 10-year metastasis-free survival rate of 91.8–99.5 % [21–23]. In our study cohort, over 50 % of the deaths were owing to factors other than the primary cancer. Such competing risks of death should be taken into account when evaluating prognosis. Recently, competing risk nomograms have been developed for sarcoma [24], breast cancer [25], prostate cancer [26], renal cell carcinoma [27], thyroid cancer [7], and head and neck cancer [8]. To our knowledge, this is the first effort to construct a competing risk nomogram for melanoma using a population-based cohort. The nomograms presented here have good discrimination ability with a high c-index of 0.85 for predicting melanoma-specific death and 0.83 for death by other causes. The calibration plot also demonstrates that the predicted probability from nomograms corresponds well with the observed CIF.

One of the greatest strengths of this study is the large cohort size and the high quality of the SEER database. The SEER dataset contains data on cancer incidence and survival collected from population-based cancer registries. The results from a population-based study are more likely to be generalizable than those from single-institute studies, which are potentially subject to selection bias [28]. Information about the cause of death in SEER data can allow us to estimate the probability of cancer-specific death based on competing risk analysis [7, 8]. Furthermore, our study cohort has more than 40,043 patients with microscopically confirmed melanoma, including information on thickness and ulceration. This sample size is sufficiently large to allow a predictive model to be built accurately.

In interpreting the results of this study, it is important to acknowledge that some variables that may be associated with prognosis are not included in the models, such as comorbidity. Although comorbidity is not included in the predictive model, we believe that age can be regarded as a proxy to offset the impact caused by the lack of comorbidity. Other limitations include the lack of a centralized review of diagnostic specimens and over 50 % of patients being classified as having other or not otherwise specified melanoma. In addition, the accuracy of the data on cause of death is an issue of concern. A study evaluating the validity of cause-of-death certification for melanoma concluded that 93 % of deaths attributable to melanoma were actually certified as being owing to melanoma [29]. Hence, we think that the bias due to mis-recording the cause of death might have been small in the current study.

Other limitations involving testing and explanation of the model and nomogram should also be mentioned here. First, the large sample size may lead to very small *p* values in statistical tests. Second, summing the predicted probability of melanoma mortality and non-melanoma mortality from nomograms may exceed one for the high-risk group [7]. Third, including a longer follow-up, as well as novel predictors, such as mitotic rate, serum lactate dehydrogenase, and comorbidity, may improve the nomogram and thereby increase model accuracy. In addition, external validation based on other populations to provide a more accurate evaluation of model performance is still needed. Finally, although our models have been demonstrated to perform well and allow us to predict the probability of death from melanoma and other causes, the predicted value does not represent the absolute accurate probability of melanoma prognosis because it is impossible to explain all of the risk factors for melanoma-specific mortality or non-cancer mortality in these models.

## Conclusion

In conclusion, in this study, we used a large population-based cohort to estimate the cumulative incidence of melanoma-specific mortality and other causes of death in patients diagnosed with melanoma. The large study cohort enabled us to build a reliable competing risk model and nomogram. Model performance was found to be good. This individualized predictive tool can be used in clinical practice to help in treatment-related decision making.

## Abbreviations

AJCC, American Joint Committee on Cancer; CIF, cumulative incidence function; ICD-O-3, *International Classification of Disease for Oncology*, Third Edition; LA, Los Angeles; NOS, not otherwise specified; _sd_HR, subdistribution hazard ratios; SEER, Surveillance Epidemiology and End Results; SF, San Francisco; SJM, San Jose-Monterey
